# 

**Published:** 1889-06-29

**Authors:** 


### NOTICE TO CORRESPONDENTS.

All MS., Letters, Books for Review, and other matters intended for the Editor, should be addressed The Editor, The Lodge, Porchester Square, London, W.

### Notice to Advertisers and Subscribers.

All Advertisements, Orders for Copies of The Hospital, and Business Communications should be addressed to The Publisher, *not to the Editor* The Hospital, 139-140, Salisbury-court, Fleet-street, E.C.

Vol. V., now ready, 4s., post free. Cases for binding, may be had of the Publisher, at 1s. 6d. each.

### To Residents, Secretaries, and Matrons.

The Editor will be glad to have the Regulations and each Report as issued by Asylums, Hospitals, Convalescent and Nursing Institutions, as well as those of other Charities, and an early copy in duplicate of all Appeals and Festival Notices, etc., addressed to *The Editor, The Lodge, Porchester-square, London, W.* Any superintendent, secretary, matron, or other chief official who regularly sends such information may be registered, on application to the Publisher, to receive free of cost a cover for binding The Hospital in half-yearly volumes.

### Amusements and Relaxation.

Second Quarterly Word Competition Commences July 6th, 1889.

### Word Competition.

Commenced April 6th, Ends June 29th.

Three Prizes of 15s., 10s., 5s., will be given for the largest number of words derived from the words set for dissection.

Proper names, abbreviations, foreign words, words of less than four letters, and repetitions are barred; plurals, and past and present participles of verbs, are allowed. Nuttall's *Standard* dictionary only to be used.

N.B.—Word dissections must be sent in WEEKLY, arranged alphabetically, with correct total affixed.

The word for dissection for this, the *Thirteenth* week of the quarter, being

"BRASSEY."

### Results of Eleventh Week.

| Names. | June 20th. | Totals. | Names. | June 20th. | Totals. |
|---|---|---|---|---|---|
| Patience | 53 | 704 | Tiny | 54 | 625 |
| Nurse Duty | 49 | 694 | N'importe Qui | 53 | 573 |
| Lightowlers | 55 | 686 | Pollie | 47 | 504 |
| Scrooge | — | 541 | W. G. R. | 40 | 518 |
| Reldas | — | 250 | Robe | — | 150 |
| Tinie | 53 | 704 | Beryl | — | 391 |
| K. G. | 50 | 655 | Daffodil | — | 89 |
| Broxbourne | 55 | 678 | A Schoolgirl | 28 | 240 |
| Lauriston | 54 | 677 | Alice R. | — | 63 |
| Spes | 50 | 655 | Belfast | — | 163 |
| Gretta | — | 224 | Gentian | — | 209 |
| Francesca | — | 476 | Hope | — | 39 |
| Heatherbell | — | 185 | Louie | — | 139 |
| Sycamore | — | 201 | Hamilton | — | 121 |
| Arctus | — | 499 | E. M. | — | 120 |
| Amicus | 50 | 657 | F. J. D. | — | 112 |
| Scratcher | — | 197 | Ladyr | — | 102 |
| Jemima | — | 138 | Surgical Nurse | — | 128 |
| Concours | — | 427 | L. Pearce | — | 32 |
| C. J. S. | — | 134 | Contentio | — | 98 |
| See-Saw | 45 | 610 | M. W. | — | 74 |
| Rosemont | - | 173 | Esperance | — | 75 |
| Jenny Wren | 43 | 430 | H. B. | — | 10 |
| Eric | — | 496 | W. G. R. (June 13th) | 11 | — |

### Notice to all Correspondents.

N.B.—All letters referring to this page which do not arrive at 139 and 140, Salisbury-court, London, E.C., by the *first post on Thursdays*, and are not addressed PRIZE EDITOR, will in future be disqualified and disregarded

Competitors can enter for *all* quarterly competitions, but no competitor may take more than *two quarterly prizes* during the year.

### Conditions.

I. Every paper sent in must be clearly written, and one side only must be used. All competitors must mark at the head of their papers the number of their competition.

II. Each paper must be signed by the author with his or her real name and address. A *nom de plume* may be added if the writer does not desire to be referred to by us by his real name. In the case of all prize-winners, however, the real name and address will be published.

III. All communications relating to prize competitions should be addressed to "The Prize Editor, The Hospital, 139 and 140, Salisbury-court, London, E.C." Competitors are requested not to include in letters, etc., to the Prize Editor communications on matters of other business. All letters must be fully prepaid.

IV. The Prize Editor of The Hospital is the sole judge of the competitions, and his decision is in all cases final and without appeal.

V. The Prize Editor reserves the right to publish any paper sent in, whether it receives a prize or not. He does not hold himself responsible for any MSS. sent, nor can he undertake to return rejected competitions.

### Scraps and Gleanings.

An outbreak of anthrax at Copenhagen is announced.

There have lately been five deaths from hydrophobia at Milan.

Purton Friendly Societies kept Hospital Sunday on June 8th.

Hospital Sunday at Savernake brought in £8. It was a wet day.

It is reputed that a woman aged 150 has just died at Meean Meer, India.

A Seaside Holiday Camp for poor London boys has been pitched at Deal.

Hull Hospital Saturday Fund amounted to £455; the best on record.

The cholera is decreasing in Madras, and affairs in Gamjan look more favourable.

The Royal Commission of Vaccination held its first meeting last Wednesday.

A farmer died last week from suffocation caused by a bee stinging him in the throat.

Mr. Lawson Tait is a member of the Association for the Total Suppression of Vivisection.

Charing Cross Hospital sports took place at Richmond. Lady Hunter presented the prizes.

Stockwell Orphanage kept the birthday of its president, the Rev. C. H. Spurgeon, on June 20th.

Carlisle Hospital Sunday Fund amounted to £874, and the Hospital Saturday Fund to £181.

The working men of Tunbridge Wells have held a meeting to organise a collection in aid of the hospitals.

The Archbishop of Cyprus was present at the Hospital Sunday Fund Service at St. Peter's, London Docks.

The friendly societies of Woolwich and neighbourhood are forming a Convalescent Home Mutual Aid Society.

The Rev. John Storrs took the chair at the annual meeting of the Gordon Hospital supporters on June 18th.

The death is announced of Mdme. Bouree, grandmother of the French Minister at Brussels, in her 101st year.

Flower Services are now becoming common in country districts, to the delight of patients in London hospital wards.

A Church Parade was held last Sunday by the friendly societies of Hampstead, in aid of the Home for Blind Children.

There were thirty applicants and five vacancies at the last meeting of the governors of Dublin Hospital for Incurables.

Deaths from diarrhœa and dysentery in London have risen from about 15 a week in May, to about 35 a week during June.

The proposed memorial to the Dowager Lady Kinnaird is to be Hospital for Women at Lucknow, officered by women doctors.

M. Dusfresne, a French chemist, who tried to bribe the managers of the French Exhibition, has been struck off the list of exhibitors.

The death of a child at Bow has been ascribed to the fact that no medical man was in attendance, the family belonging to the Peculiar People.

The committee of the Royal Humane Society on Saturday announced the award of twenty medals for saving life from drowning. Two of these were silver medals.

Another lively meeting was held at the Suffolk General Hospital on June 19th, which ended in the medical staff becoming members of a committee for revising the present rules.

The Church Society for Providing Homes for Waifs and Strays has received a donation of 100 guineas from the Bishop of London from a fund put at his disposal for such purposes.

It is said that more money has been spent by the United States Government in the investigation of the diseases which affect swine than of those which affect the human species.

Prince George of Wales will lay the foundation-stone of the new branch hospital of the Seamen's Hospital Society (late "Dreadnought"), at the Albert and Victoria Docks, on the 17th July.

The Duchess of Albany paid a visit to the Soldiers' Daughters' Home at Hampstead, on the occasion of the thirty-fourth anniversary general meeting of that institution, and distributed the prizes.

It is stated that the German Government is contemplating a measure requiring all chemists who undertake food analyses to provide themselves with a diploma to be obtained by passing a State examination.

The Princess of Wales has consented to be present at a fancy sale and concert to be held at Surrey House, 7, Hyde Park-place, by the kind permission of Mr. and Mrs. Cyril Flower, to-day, from 3 to 7.30 p.m., in aid of Miss Ada Leigh's homes in Paris.

The Prince of Wales has fixed Wednesday, July 24th, as the date for laying the memorial-stone of the new building for the Samaritan Free Hospital, now in course of erection in the Marylebone-road. He will be accompanied by the Princess of Wales.

In the Department of the Rhone it is said that children under one year form 48 per cent. of the deaths, in Ardeche as much as 74 per cent. One reason of this enormous infant mortality is that so many French mothers try to bring up their children on artificial food.

The *Charity Record* states that at the National Hospital for the Paralysed, Queen's-square, it is proposed to establish post-graduate classes, wherein those who have received their imprimatur as being able to treat general diseases may specially study the many special and nervous diseases.

